# Nurses' Relational Leadership Struggles on Positioning in Strategic Hospital Crisis Management: A Qualitative Interpretive Study

**DOI:** 10.1155/2024/9212508

**Published:** 2024-10-12

**Authors:** Arjan Verhoeven, Erik van de Loo, Henri Marres, Pieterbas Lalleman

**Affiliations:** ^1^Radboud University Medical Center, Otorhinolaryngology, Head and Neck Surgery, Nijmegen, Netherlands; ^2^Department of Organisational Behaviour, INSEAD Europe Campus, Fontainebleau, France; ^3^Research Group Person-Centredness in an Aging Society, Fontys University of Applied Sciences, Eindhoven, Noord-Brabant, Netherlands

## Abstract

**Aim(s):** To understand how nurses experience their positioning amidst hospital crises.

**Background:** Nursing leadership literature is predominantly focused on the skills and competencies of nurses and less on the relations in practice with nurses. Nurses are often valued for bedside care but are overlooked in strategic decision-making during crises. Foundational research emphasizes the need for nurses' equal participation in interprofessional healthcare practices and governance.

**Methods:** We conducted a qualitative interpretive interview and focus group study, amidst the COVID-19 crisis. We interviewed 64 chairs of nurse councils and deepened our understanding of our initial findings in four focus groups with 34 participants.

**Results:** Nurses differ widely on (a) what is important to them in crisis management, (b) how they can contribute to crisis management, and (c) how they value their involvement or lack of it. Furthermore, we uncovered three relational leadership struggles for nurses concerning (1) navigating, (2) positioning, and (3) collaborating, in crisis management structures.

**Conclusion:** The ailing positioning and representation of nurses in crisis management result from their limited participation in strategic decision-making, and the lack of intervention on this by board members, physicians, and managers.

**Implications for Nursing Management:** This study highlights the need for agents such as board members, managers, physicians, and nurses themselves to create clear frameworks and policies that define nurses' roles in crisis situations, emphasizing the importance of addressing power dynamics and enhancing communication and collaboration in hospital settings. Effective crisis management requires involving nurses from the start, providing regular training, and promoting a more equal approach to teamwork. Understanding relational leadership and its impact during crises can empower nurses and improve overall hospital crisis response.

## 1. Introduction

Nursing leadership plays a crucial role in ensuring and providing quality care during hospital crises [1, 2]. According to Kim [3], nursing leadership in crisis is *“clear, fast, and frank communication and a high degree of collaboration; sharing of information; decision-making and fair prioritization; building trust; and competency”* (p. 4). However, recent research indicates that professional nursing governance structures, which empower nursing leadership, are often overlooked in strategic decision-making during such crises [4]. Most nursing leadership literature focuses on individual attributes such as style, traits, and skills [5–7], which contrasts with the growing emphasis on leadership as a collective, relational, and ongoing process [8].

Healthcare crises act as a “stress test” for relational and collective leadership within hospitals [9] because these situations demand collective awareness [4], collaborative efforts [10], rapid decision-making [2], and cooperation across organizational and professional boundaries [11]. As Martini et al. [12] highlighted, nurses need to navigate relationships, influence social structures, and gain the trust and respect of key stakeholders, such as board members, managers, and physicians, who dominate strategic organizing during crises.

Positioning in the strategic management of hospital crises is a highly interdependent and relational leadership task [13, 14]. Despite this, clinical nursing leadership remains primarily focused on bedside care [7, 15], which suppresses broader professional nursing organizing work [16, 17]. This narrow focus hinders the necessary relational work for collective strategic positioning in crisis management [18].

Therefore, there is a need for a practical and relational approach to nursing leadership [1, 19]. This approach should complement the existing literature and practices, particularly regarding the relevance of relational nursing leadership during crises. This study, therefore, used a qualitative interpretive methodology to explore nurses' experiences during the COVID-19 crisis, focusing on relational leadership and crisis management. Findings revealed significant differences in nurses' perspectives on crisis management and highlighted key leadership struggles in navigating, positioning, and collaborating. The study's findings can help agents in hospitals to be better prepared for the positioning of nurses and collaboration between professionals. According to Kvist, Seitovirta, and Nurmeksela [2], such collaboration leads to better decision-making and empowerment of all agents involved.

## 2. Background

### 2.1. Relational Leadership Processes in Crises

In the nursing leadership literature, there has been an overreliance on the transformational model of leadership [20] and the “focal leader” [21]. This focus has emphasized skills and traits [15], portraying nursing leadership mainly as “effective leadership” [12]. However, following the practice [22] and relational [23] turn, relationships have received increased attention in nursing leadership and governance research [13, 24]. Despite this shift, the literature still primarily focuses on interpersonal relationships [13], relational skills [25], and leadership styles [5]. According to Uhl-Bien [14], such approaches to relations are entitative, focus on individual agency, and consider agents involved as *“independent, discrete entities”* [14]. A relational, processual approach departs from “*processes, not persons, and views persons, leadership and other relational realities as* made *in processes”* (emphasis in original, [14]). Relational leadership is then a plural and collective form of leadership [14], as are distributive, shared and leadership-as-practice [8]. From this perspective, leadership is seen as an *“iterative and messy social process that is shaped by interactions with others”* [14, 26]. This fits with the understanding processes of positioning by nurses, and other agents, in crisis settings [27].

### 2.2. Positioning of Nurses in Crises

The frames of nursing as a powerless [28] and exceptional profession [29] are paradoxical and have historically led to *“circumstances of marginalization, invisibility, and gender biases”* [29]. Furthermore, the acknowledgment of nurses as professionals has not led to a change in the (organizational) positioning of the nursing profession [25]. Consequently, the positioning of nurses in the recent pandemic was poor and is reviewed critically [30]. According to Porter-O'Grady [7], nurses have widely received appreciation for their bedside work but have been neglected as professional experts in strategic decision-making during this crisis.

The work of, among others, Porter-O'Grady and Finnigan [31] laid the foundation for research on such strategic, shared, and professional positioning and governance of nurses. This work created a structure for positioning of *“(…) professional nurses as an equitable partner in translating clinical principles into interprofessional practices and relationships across the healthcare continuum.”* [4]. According to Porter-O'Grady and Pappas [4], professional governance encompasses *“(…) decisions about practice, quality, competence, and knowledge. (…)* (and these) *are the substance of the work of a profession and, by both regulation and disciplinary mandate, are drivers of the life of the professions in the world.”* [4]. However, these governance structures affect not only the positioning of nurses alone but also the positioning of other agents (i.e., board members, managers, and physicians) in hospitals. This interdependence seems to be overlooked in the professional governance literature and practice. Moreover, this neglect of interdependence of nurses and others hinders the involvement of nurses in strategic decision-making [32], and this is not the responsibility of nurses alone [2, 28]. However, how nurses themselves enact their (relational) leadership, work with interdependence, and belief in the importance of their voice and positioning are essential in strategic crisis management [2, 33]. Our study was, therefore, led by the question of *how do nurses perceive their positioning in crisis management, from a relational leadership perspective?*

## 3. Methods

### 3.1. Study Design

We used a qualitative interpretive approach [34] and used individual and focus group interviews to capture *“the meaning of lived experience”* [35]. This approach and these methods fit with the aim of our study to enhance the understanding of how nurses in Dutch hospitals, amidst COVID-19 pandemic, perceived their positioning in crisis management.

### 3.2. Context of Crisis in the Netherlands in Early 2020

On the 11th of March, the World Health Organization (WHO) declared the COVID-19 outbreak a pandemic [36]. Late March, the crisis reached a peak in the Netherlands due to the shortage of personal protection equipment (e.g., high-quality face masks and gloves), (specialized) nurses, intensive care unit (ICU) beds, and ventilators [10]. The pressure, however, was not evenly spread over all 72 Dutch hospitals as mainly the hospitals in the South of the Netherlands were overrun by very sick and dying patients. At this time, the Dutch society, as most of the world, was in a state of crisis. Internationally, the general opinion was that nurses played a crucial role in keeping the healthcare infrastructure afloat [10].

### 3.3. Data Collection

**Figure 1:**
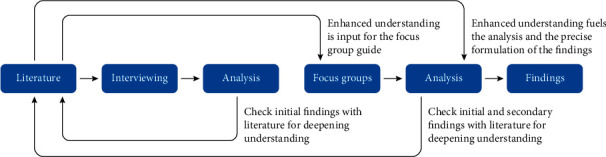
Visualization of the research process.

The research was conducted between April 2020 and December 2021. In April 2020, interviews (*N* = 46) and email exchanges (*N* = 18) with nurses took place. In addition, based on the interview data, four-member check focus groups [37], with 34 participants, were conducted in June 2021.  Figure 1 visualizes this research process.

### 3.4. Interviews With Nurses (April 2020)

Via a database from the Dutch Nurses Association (i.e., V&VN), we emailed the chairs of the nurse councils of all 72 Dutch hospitals. These chairs were registered nurses (RNs), and worked, besides their chairing work, on the wards, during data collection. Therefore, in this paper, we refer to them as nurses.

We made an interview topic guide based on nurses' first-hand experiences in crisis management and emailed the interview questions (see Appendix A) prior to the interviews. We interviewed 46 nurses via (video)calls, in a 13-day timeframe. The other 18 nurses answered our questions by responding to our email because they were unable to be available for our interview.

The interviews were conducted by the first author and last author, and eight other interviewers. All interviewers are knowledgeable about the work of nurses and specificities of (Dutch) hospital governance. The interviews were guided by eleven open-ended questions regarding the role, involvement in hospital crisis management, voice and leadership, relationality with other organizational actors, and foreseeable involvement of nurses in the postcrisis organization of care processes. All participants were asked to score their involvement in strategic crisis management on a scale from 1 (*no position*) to 10 (*excellent position*) and to elaborate on their score. This was done to elicit [38] a discussion on what nurses experience as positioning (e.g., “Why do you score a six? What would have made it a seven?”).

In four of the 46 interviews (9%), no score was given, or was, in hindsight, ill substantiated. In addition, in four of the emails (22%), the explanation of the score was insufficient. Therefore, 56 useable scores were included as data.

All interviews were audio-recorded (> 21 h) and transcribed (466 pages). The average interview duration was 28 min. Per interview, a summary was made directly after the interview and sent to the interviewee for a check. Informed consent was initially audio-recorded and afterward retrieved via emails.  Table 1 gives an overview of the interview data per type of hospital.

### 3.5. Focus Groups With Nurses and Various Agents (June 2021)

After the initial analysis of the interviews, we found that the interdependence between nurses, managers, physicians, and board members, in crisis management, stood out. We therefore emailed all our interviewees in January 2021 and asked them to participate in a focus group interview and to invite representatives of these groups for focus group discussions on the positioning of nursing in crisis from different perspectives. We received more response than anticipated. We, therefore, added a fourth focus group. In total, 34 participants from 12 general hospitals and three academic medical centers participated in the focus groups, in June 2021. Topics were based on the analysis of the interviews: collaboration, decision-making, learning, structure and governance, and positioning (see Appendix B). Because of the relational nature of these topics and to elicit [38] a dialog between various involved agents, we composed each group with a mix of board members, managers, nurses, and physicians from different organizations. The aim was to make the groups as diverse as possible.

All focus groups took place via ZOOM© and were chaired and observed by the first author (AV) and last author (PB). The observations were discussed directly after each focus group and led to brief reports, which were used in the following focus group meetings. All focus groups were video- and audio-recorded and transcribed. The recordings, transcripts, and discussion reports (10 pages) were used for analysis. All participants gave informed consent prior to the focus groups. This study is compliant with the COREQ-standard [39].  Table 2 shows the data collected with the focus groups.

### 3.6. Data Analysis

We familiarized ourselves with the audio and video recordings, the transcripts, and the observation reports. We used Atlas.ti© software for the coding process. Research memos and timestamps logged the process of the researchers during familiarization and coding for reflexivity and rigor. This is consistent with the interpretive description methodology [40] and led to insights into how crisis management was structured in different hospitals.

Furthermore, with the use of Microsoft Excel©, we plotted the process of involvement in crisis management and the different scores that the interviewees gave to their involvement. We ran different analyses in Excel to see the relations between the type of hospital, the moment of involvement, and the given valuing score for involvement. We read the transcripts to filter on understanding the value of the scores given. In eight interviews and emails, the scores were missing or ill substantiated. We eliminated these from the data. Understanding the background of the scores given led to plotting the scores on a three-part scale in *low* (1–5), *medium* (6–8), and *high* (9-10) scores. This leads to a better representation of the scores given and the explanation thereof given by the participants. An overview is enclosed in Appendix C.

Furthermore, iterative rounds through the data using Atlas.ti© and discussions on our findings enabled us to deepen the analysis and reach beyond initial themes [41]. Moreover, the influence of the talk by the board members on positioning and voicing stood out in the analysis of the focus groups. We, therefore, ran a separate, additional analysis on this part of the data and reported on this in a separate paper. This paper focused on the perspective of board members [27].

From the analysis of the interviews and focus groups emerged three relational leadership struggles specifically for nurses. In this paper, we report on these struggles.

## 4. Findings

We present our findings, first, by unpacking the organization of crisis management in the hospitals. Second, we show the data on how nurses perceived their involvement in crisis management, and, third, we present three relational leadership struggles for nurses that emerged as such from our abductive analysis of the interviews and focus groups.

### 4.1. Organizing Crisis Management

In the interviews and focus groups, much was said about how the crisis management was structured in different hospitals. In general, the crisis management structures consisted of operational teams and a strategic crisis management team. The operational teams were structured around topics such as safety, deployment of personnel, and collaboration with other institutions. They prepared policy and advised the strategic crisis management team on decisions to take. The strategic crisis management team was leading and took decisions that were executed by the operational teams and/or other organizational units.

We learned that this formal structure developed over time. In the focus groups, participants stated that at the start of the crisis (March 2020), there was a strict *“command structure”* with rules and hierarchy determining decision-making. From July 2020 onwards, this developed into what a participant called a *“deliberation structure.”* This was often referred to as a diffuse situation because there was a crisis management structure, but there was no crisis anymore in the sense that COVID no longer had the characteristics of a crisis (unanticipated, ambiguous event that is a significant threat). The crisis management structure, however, held priority over the daily (hierarchical) organizational management structure. In the focus group discussion, this role's ambiguity was referred to as hindering clear positioning of groups, such as nurses.

### 4.2. Perceived Involvement of Nurses in Crisis Management

Interviewees differed widely on how they perceived and valued the involvement of nurses in crisis management.

In only four hospitals, nurses were directly involved in crisis management from the early start. In these cases, the nurse council (hereafter council) was invited to participate by an executive hospital board member (hereafter board member) in the strategic crisis management team.

Twenty interviewees reported that they were involved in crisis management after they themselves requested to be structurally involved. These requests were all done via a board member. In most cases, these nurses were referred by the board member to the chair of the strategic crisis management team for their request to participate.

No less than forty interviewees reported that they were not involved in crisis management in their hospitals. Of these, in 14 hospitals, a request by the nurses for the involvement toward the chair of the strategic crisis management team or directly to the board was either declined or ignored. On two occasions, the reason for declining participation was the hospital's crisis management policy that participation bodies (such as the council) were not seen as necessary to bring in specific expertise because other frontline professionals were given the task of providing nursing expertise. Councils regarded this a valid and acceptable decline because nursing expertise was believed to be secured in the crisis management structure by either frontline nurses or nurse managers with adequate expertise.

Furthermore, 13 nurses reported that no or little involvement was acceptable to them if timely and adequate information was given or when nursing expertise was guaranteed.

Three interviewed nurses stated that nursing expertise was absent in crisis management and their request for involvement in crisis management was declined. In these cases, either a management representative involved was considered to be sufficient by those responsible for crisis management or nurses were not seen as essential in crisis management. Both reasons were not acceptable according to the interviewees. The former—commitment of a management representative as sufficient to bring in the nursing expertise—was to the interviewees striking. One of the interviewees stated on this:

*“So, it is a manager that represents nurses in crisis management. (…) I had a discussion on this topic with a physician. He told me “What are you whining about? Lisa is at that table representing you, right!?” And then I said, “Well, she is not working as a nurse, now, is she?”. On which he replied, “Well, she was once, wasn't she?” And then I asked him if this would hold for the medical manager (who is not practicing medicine for some time)? And he replied, “No, of course not, but this is different for nurses, is it not?” It is these beliefs that are persistent and to me really disappointing. And he (doctor) is not the exception.” (Interview nurse, hospital 33)*

### 4.3. Scoring of Satisfaction on Involvement in Crisis Management

**Figure 2:**
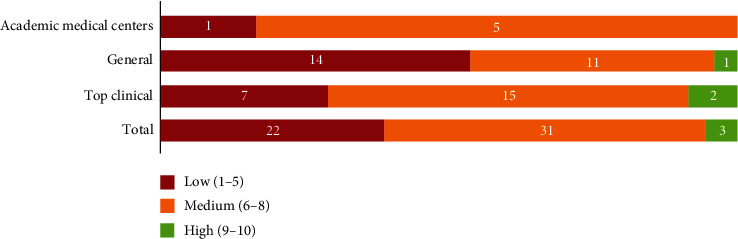
Scoring of satisfaction on involvement in crisis management.

In our interviews, nurses scored and elaborated on their involvement in crisis management. Of the 56 scores, there were three “high” scores. Thirty-one nurses gave their involvement a “medium” score, and 22 valued this as “low.” The distribution of scores across academic, general, and top clinical hospitals is shown in Figure 2.

Access to information, being informed, level of participation in crisis management, and being heard by the board were the most influential factors for the scores (e.g., valuable information led to a higher score, and no access to the board led to a lower score).

We learned that the score of the involvement in crisis management and the actual involvement differed. What stood out, for instance, was that the interviewees of academic medical centers mostly scored their involvement as “medium,” but none were involved in the strategic crisis management team. Their score was substantiated with arguments referring to how they were informed on crisis management, how other involvement of nursing expertise was organized in their hospitals, and when appropriate the nurse council was consulted as a participation body. One of the interviewees stated on this:

*“No, we as council are not involvement in crisis management. When we realized this, we checked with the nurse managers—who have been well organized for the last two years—whether they were involved, and when we found out that they were we let it be. (…) For us as council it is most important that wards are prepared for COVID and non-COVID, adequately, and with the right staff. (…) We are regularly informed by the board and at times consulted on council topics. This is sufficient, for now.” (Interview nurse, hospital 6)*

Therefore, being informed stood out as why a lack of direct involvement of our participant was acceptable, at that moment in the crisis. Three paths of information were distinguished by our interviewees: first, direct information from the board, second, information via members of the council who also worked as nurse managers and participated in crisis management in their management role, and third, information reaching the council informally from co-workers who were in diverse ways involved in or informed about crisis management. On some occasions, the informal information also came from a board member.

From the interviewees we learn that in some cases, the absence of nursing representation in (initial) crisis management is due to “simple” facts such as that nurses were missing on the lists of people or bodies that need to be informed, consulted, or involved in the case of emergency or crisis in mandatory and standardized organizational emergency plans. In contrast, according to the interviewees, management and medical representatives are always mentioned in these plans.

In other cases, not involving councils or securing nursing expertise at strategic crisis management level in other ways was a deliberate choice by crisis management or boards. This was mostly because nurses were seen as being of more added value in an operational team. There were also cases wherein councils themselves decided that strategic crisis management was for others to manage and that nurses needed to be on the front line of care, as we learn from the quote below:

*“Well, when we are asked last week which strategy, we as council had chosen for our involvement my first thought was; ‘Did we do the right thing?'. But you know, at that moment when the corona crisis broke out in full force and that it, uhm, that the whole hospital was turned upside down and became a, uhm, corona hospital. Well then, we went with the flow. We rolled up our sleeves and went to work. We did not reflect on our position or strategy. We just went in.” (Interview nurse, hospital 70)*

### 4.4. Relational Leadership Struggles of Voicing and Positioning in Crisis

In our analysis of the interviews and focus groups, we found three distinct relational leadership struggles for nurses: navigating, positioning, and collaborating in times of crisis. We coin our findings as struggles to stress the perceived complexity by nurses of relational leadership in crisis. We elaborate on each of these three struggles and highlight these with quotes.

### 4.5. Navigating

The struggle of navigating was part of the crisis but was, for the interviewees, intensified because of decisions made at the start of the crisis and the limited possibilities given by other agents to restore this ambiguity on tasks, roles, and positioning.

For all working in a crisis management structure, this was new, and there was no guideline on who was supposed to do what in this situation. This led to insecurity and ambiguity. Furthermore, the scale of the crisis and the call to nurses to contribute to the frontline of care made navigating in what was wise to do complex. In addition, as one of the interviewees stated, *“there was no manual.”*

Part of this struggle was the complex decisions that some nurses felt forced to make between organizing work and direct, frontline nursing work. Overtaken by the crisis and in hast, most decided to contribute to direct care. This led in numerous hospitals to unmanned councils and was seen as a *“surprising move,”* by board members and managers in our focus groups. However, we learned that they took no action on this “surprise.” One of the nurses said

*“So, no, we were not clear in why we wanted to be in a operational team, or that we should be in the strategic crisis management team. (…) No, we did not make that clear, and honestly, I don't think we were able to live up to that either. (…) But in hindsight, I wish we had been more persistent. It would have been a lot of work but it would have helped us (nurses) enormously in staying in tune with the (crisis) organization. Now that we are not in we can't keep up and are not informed well enough to do our council tasks.” (Interview nurse, hospital 20)*

In this quote, the struggle for navigating expectations becomes apparent. Furthermore, it highlights the interdependencies between agents on information and expectations.

However, time to consider the task at hand and the role of the council in crisis was absent, according to the interviewees. According to one of the nurses, this led to complications in the role and task navigation later in the crisis. He stated

*“Well, my personal experience is, that when we went to the chair of the board earlier this week to pass on signals we received—negative signals—on flexpool and other topics, well, then, he sighs, so to speak. So, well, of course it is not nice to receive these negative signals from your nurses, but I do feel this is our task here. (…) And, this is important; be clear on the task you take up, so you don't run too fast for others to understand what you are doing.” (Interview nurse, hospital 20)*

It becomes clear that relating and attuning was important for nurses to navigate the channels of information and tasks in crisis and that such processes come with responsibilities for other agents, such as the chair of the board in the quote above.

### 4.6. Positioning

Positioning highlights the interdependencies between agents and is therefore part of relational leadership work. Positioning also affects how, for instance, nurses are informed and are able to contribute to organizing work in crisis.

The struggle for positioning unfolds between how the council presents itself and how the council is perceived and positioned by other agents (i.e., board members, managers, and physicians) and the nurses on the wards of the hospital. From one of the emails we received in response to our request for an interview, we learned

*“I find it hard to determine how satisfied we are with involvement. I would score it a 5 because we are not involved. Then again there is medical and nursing expertise represented in crisis management. When we proposed to the board member that we would participate in crisis management she replied that this is not a job for participation bodies such as the council because there is a need for direct care expertise and therefore front line professionals.” (Email nurse, hospital 45)*

Although all interviewed nurses were also working as nurses on the wards, the quote highlights that the positioning of the council and the possible contribution to crisis management are unclear to the board member, and this leads to a certain positioning on which the nurse has little influence.

In contrast with nurses, we learned that formal management roles of physicians led, automatically, to positioning in (strategic) crisis management structures. However, although some of our nurse interviewees also had such *“double roles”* (i.e., being a member of the council and being a nurse manager), our interviewees expressed concerns for how the crisis management structure depended on the general quality of nursing expertise from managers. One interviewee informed us as follows:

*“The managers are not nurses, anymore, if you ask me. But they participate in the crisis management structure as nurse representative, and of course they have some knowledge, but in this way nursing expertise is missing, in my opinion.” (Interview nurse, hospital 33)*

The interaction between nurses at the wards and managers of nurses was also linked to exchanging information as part of positioning. One of the interviewees stated that contribution was central to the goal of positioning:

*“We are well connected to the work floor; a representative of each ward is represented in our council. So, this makes it easy to discuss and check ‘are things taken care of, or are we missing anything', positive or negative. Also, positive things can be informative to encourage to stay on a certain track.” (Interview nurse, hospital 13)*

This quote shows not only the goal of contribution but also how positioning opens channels of information. These channels can lead to collaboration, but to an extent, these also constitute the information dependency of nurses on other agents. Our interviewees informed us that they were mostly okay with not being part of the (full) crisis management structure but that they experienced unwanted information dependency. This conflicted with the tenet of the council to be informed and able to advise or mobilize based on an informed status.

### 4.7. Collaborating

Collaborating was seen as a process influenced by expectations and the interaction between organizational and crisis management structures. The ambiguity of the context (i.e., crisis and confusing structures) influenced processes of collaborating and had a backlash on the possibilities for nurses to position themselves because it was unclear who had what position.

Furthermore, in the interviews and in the focus groups, it became apparent that the meaning of collaborating is influenced by the perspective (e.g., nurse, physician, manager, and board member), context (e.g., crisis and noncrisis), and hierarchy (e.g., subordinates versus “superordinates”). While managers and board members (i.e., “superordinates”) often named the constructive and pleasant collaboration with nurses, nurses highlighted the struggle to find balance between their professional standards and autonomy and what they were ordered to do in the crisis setting by those higher in the hierarchy.

Furthermore, in the focus groups, we learned that at the beginning of the crisis, there was no room for discussions on positioning and the ideal organization. There was a strict hierarchy, focused on what participants called *“military-like, command and control*.*”* The focus was on survival, and the limited space for other kinds of communication appeared to be difficult for nurses.

Furthermore, we learned that attuning between agents is essential for collaborating in crisis, and nurses found themselves as not seen. As a nurse told us

*“But honestly, the board nor the involved managers ever asked us to share our knowledge. Not at all. And we keep telling them ‘Hello, here we are, we want to help, and it also concerns nurses and not only physicians, to know how things are going, what decisions are made, and which effects this resort'.” (Interview nurse, hospital 25)*

Furthermore, examples were given by our participants on certain assignments from the top of the crisis management hierarchy that were in contrast with ethics and good practice in nursing such as the limiting of visiting hours for family. This led to tensions that fueled the importance of hierarchical positioning and therewith complicated collaborating.

## 5. Discussion

This paper contributes to the nursing leadership literature by examining how nurses struggle with their positioning in strategic crisis management during crises. Our study reveals that nurses are often positioned in practical roles rather than strategic ones, partly due to their own choices that limit their involvement in decision-making. Additionally, our findings indicate that interdependencies among various stakeholders—such as nurses, board members, managers, and physicians—are often neglected in practice, which undermines effective collaboration during crises. Board members and managers typically see nurses as primarily useful for practical crisis preparation, without recognizing the importance of addressing these interdependencies [27]. Furthermore, our study shows that positioning during crises is intertwined with hierarchical power dynamics and the ability to express one's voice. While hierarchy and enabling nurse positioning can coexist, most nurses reported their involvement in crisis management as “medium” (55%) or “low” (39%).

Below, we will first discuss the practical positioning of nurses during crises, then examine the neglect of interdependencies, and finally reflect on the tension between hierarchical structures and the positioning and voicing of nurses.

### 5.1. Positioning of Nurses as Practical

The importance of involving nurses in strategic decision-making has been well researched [42] and is seen as crucial for the postpandemic future of healthcare [43]. In our study, nurses and other agents viewed informing and being informed as acts of involvement. However, being informed is not the same as being positioned to influence strategic decision-making, which can result in poor positioning of nurses in crisis management.

In recent work, Porter-O'Grady [7] states that, *“much of the structure, systemic relationships, leadership approaches, and organizational infrastructures relate to nurses predominantly as a blue-collar employee-dependent work group.”* [7]. This indicates that the inadequate positioning of nurses during crises is not incidental but systemic. Therefore, all agents, including nurses, must work to change the narrative and agency of nurses to ensure their effective positioning in crisis management [28].

### 5.2. Neglecting Interdependencies

The relational struggles, central in this paper, were portrayed as struggles of nurses in the interviews and focus groups. However, such struggles are not of nurses but are systemic, relational, and social [7] and highlight interdependence between agents. Seen as such, these struggle are of board members, managers, and physicians as well [27]. Furthermore, although interdependence between professionals is inherent to healthcare and nursing [44], the literature on interdependence in healthcare is scarce [45]. Moreover, the dominant focus in the interdependence literature is on task interdependence (e.g., what is done) and not on agent interdependence (e.g., who does what) [46]. Agent interdependence encompasses goal setting and expertise between agents and is, therefore, relational. The little attention to agent interdependence is, according to Raveendran and Silvestri and Gulati [46], the consequence of ignoring the shifting complexity of work. For nursing, this involves a lack of acknowledgment of the profession's breadth [16, 47, 48]. Recognizing the full scope of nursing by others is essential for effective and interdependent collaboration. In other words, if an agent does not recognize another agent as relevant to their task, goal, or knowledge, they cannot begin to cooperate from an interdependent perspective. For example, Verhoeven et al. [27] demonstrated that when the board members acknowledge and consistently position the organizing expertise of nurses, they can work more effectively on quality and safety goals (goal interdependence) and innovate work processes to address staff shortages (knowledge interdependence). These interactions are relational and not typically outlined in job descriptions [13].

### 5.3. Hierarchy and Voice

In our study, we found that positioning during crises is relational and intertwined with hierarchical power and influences the ability to use one's voice. Furthermore, although all agents expressed a willingness to collaborate, those with hierarchical power—such as board members and managers—often seemed unaware of their power and its effects on the voicing of others [27]. Voicing is essential for unlocking the knowledge of all occupational groups, which is key to organizational performance [19]. However, agents with hierarchical power sometimes limit the voicing and agency of nurses [28, 49]. In the interviews, we found that especially in crises, the influence is blurred and limited due to high stakes and time constraints. In line with this, Martini et al. [12] stated that for empowerment, agents need to get *“wired in”* (p. 8). Therefore, agents need to navigate relations in the complexity of practice. This can function as a counterbalance to hierarchical power. Navigating is then dependent not only on skills, competence, or hierarchy but even more on how social structures work and how access to the relevant “tables” is organized in strategic crisis management and decision-making [36].

Our study shows that hierarchy and the enabling of positioning should go together. They do not exclude each other. A relevant future avenue for research is, therefore, to study how power structures, from a relational leadership perspective, shift across organizational boundaries [11] and how these interact with the positioning and voicing of interdependent agents, such as nurses and board members. It is, therefore, important that nursing leadership research finally embraces relational leadership [24] as an addition to the dominant perspective of individual skill and trait. Understanding relationships better in practice is crucial for nurses—and board members and managers—to better position nurses as organizing professionals [50, 51].

### 5.4. Implications

This study contributes to nursing management by emphasizing the need for clear frameworks that define roles and expectations for nurses in crisis situations. We underscore the importance of addressing power dynamics, role positioning, and representation within organizational structures, particularly in crisis settings. Organizational behavior and power dynamic theories should consider these aspects that ensure nurses have a defined and respected role during crises.

Furthermore, hospital management should prioritize policies that ensure nurses are actively included in crisis management teams from the outset. Developing effective communication strategies is essential to ensure that nurses receive timely and accurate information, which includes establishing direct lines of communication among all team members involved in crisis response. Regular crisis management training programs are also crucial. These programs should equip all team members, including nurses, with the necessary skills and knowledge to perform their roles effectively during a crisis. Training should also address how to manage and reduce hierarchical tensions that may hinder collaboration and should promote a more egalitarian approach to teamwork.

Moreover, it is important to explore the impact of hierarchical structures on collaboration and develop models that facilitate better interprofessional teamwork, particularly under crisis conditions. Understanding how relational leadership operates during crises and recognizing the interdependencies between different agents, including nurses, are vital for effective crisis management in hospital settings. By implementing these strategies, hospital management can foster an environment where nurses are empowered and can contribute fully to crisis response efforts.

## 6. Conclusions

The context of crisis entails uncertainty and ambiguity, and this has a backlash on the positioning of nurses in hospitals. Our study shows that positioning of nurses is mostly approached from the frontline work of nurses and disregards their invisible, strategic organizing work in times of crisis. This was, in part, due to how nurses themselves, in the tense crisis context, took position and prioritized caring work over strategic organizing work. However, other agents in the crisis management structures turned a blind eye to the lacking strategic involvement of nurses too. Nurses felt, therefore, at best, engaged, and at worst, not involved. Therefore, the workings in strategic crisis management did not allow for the full blending of professional nursing work and strategic crisis management, and this hindered the interdependent strategic decision-making in crisis. Nurses experience such exclusion as problematic, and this fuels the imperfect positioning of nursing in crisis management.

### 6.1. Limitations and Future Research

In this study, we used interviews and focus group meetings to discuss the processes of voicing and positioning. Although we validated our findings through member checking [37] and adhered to quality measures for qualitative interpretative research [34], we did not observe these processes in situ. Practice-oriented and ethnographic methods can provide insights into these real-time processes [12].

Additionally, we collected data over a 13-day period during an unprecedented crisis. With 10 interviewers, we used telephone, video calls, and emails to conduct the interviews. This combination may have introduced unwanted variation in data collection, impacting the study's rigor as we could not return to the field. While our study focused on intraorganizational leadership processes in hospitals during crises, further research is needed on the external factors influencing strategic choices by managers and board members [33]. Understanding how they translate policies and political decisions into their collaborative work with nurses will benefit all agents involved in strategic care organization.

## Figures and Tables

**Table 1:** Participants and data interviews.

**Type of hospital**	**# Participating hospitals**	**# Interviews included**	**# Emails included**	**Duration (# min)**	**# Pages transcript**	**# Useable scores**
AMC^∗^	8	8	0	252	85	6
General	29	19	10	457	167	26
Top clinical^∗∗^	27	19	8	603	214	24
Total	64	46	18	1312	466	56

*Note:* ^∗^Due to a merger, there are seven academic medical centers (AMC) in the Netherlands since 2018. Nevertheless, the nurse councils were not merged at the time of the interviews, and both were interviewed for our study, hence the eighth AMC entry in our dataset. ^∗∗^Besides academic and general, there are also the so-called top clinical hospitals (26) in the Netherlands. These hospitals have a more specialized infrastructure for complex care than general hospitals (see https://www.stz.nl/over-ons/stz-ziekenhuizen/, visited on the 5th of February 2023). Antoni van Leeuwenhoek and Princes Maxima Centre for (child) oncology are specialized hospitals and are in this study labeled “top clinical” to warrant anonymity.

**Table 2:** Participants and data focus groups.

**Focus group**	**# Different participating hospitals**	**# Participants**	**Board member**	**Manager**	**Nurse**	**Physician**	**Duration (# min)**	**# Pages transcript**
1	7	7	1	1	4	1	100	24
2	8	9	2	2	3	2	94	22
3	7	10	2	1	4	3	105	24
4	8	8	2	2	3	1	99	22
Total	15	34	7	6	14	7	398	92

**Table 3:** Topic guide focus group meetings.

**Topic guide focus group meetings—collaboration in times of crisis**				
**Central question: How do nurses, management, physicians and members of executive hospital boards (BM) perceive their collaboration in crisis (March 2020–June 2021)?**				
**#**	**Theme**	**Intro**	**Question/statement**	**Subquestion/subtheme**
0	Opening	Showing video of news item—BM states nurse is directly involved in crisis management and directly after a nurse reports that she is not involved	What do you see in this news clip and how can you relate this to your own practice?	What does it mean that people perceive a situation (or involvement) differently? How is this for managers, and for physicians (how are not in the clip)?
1	Collaboration	In crisis collaboration is crucial. This is also about trust, knowing each other and relating. Different interests, connection, and power	How do you look back at the collaboration in crisis between nurses, physicians, managers, and BM?	What were successes and what is there to improve? What does this mean for the coming period? How do perspectives of management and work floor align or conflict? What were troublesome topics?
2	Decision	A crisis demands swift decision-making. However, sometimes it is better to be thorough than swift	How is decision-making in crisis organized in your hospital and how are relevant perspectives involved?	How is the process of decision-making perceived? Who is responsible for this process? What is different in crisis?
3	Position	In our interviews in May 2020 on the positioning of nurses and nurse councils (as representatives of nurses) we learned positioning and involvement was poor	How are nurses positioned in crisis in your hospital? What was the role of other organizational groups in this positioning? How do you reflect on this positioning?	How did positioning of nurses develop in time? What changed? If any, what caused the change?
4	Developing/learning	We are now (June 2021) in a somewhat quite phase. What does the recent experience mean for collaboration and care in the coming times?	What are the learnings from recent experiences concerning collaboration in crisis? Working with different perspectives	
5	Structure	Structure is seen as a symbol of collaboration. In our interviews in 2020 the crisis structure was portrayed as such. Structure as a vehicle of collaboration, network, knowing and relating	How is de crisis management structure used in your hospital, and is it seen as supportive or hindering for collaboration?	How did organizational structure and crisis structure work together? What was the role of the informal structure? How was highest in command in crisis management?

**Table 4:** Overview of involvement score and start according to (C)NC, per type of hospital, and data collection method.

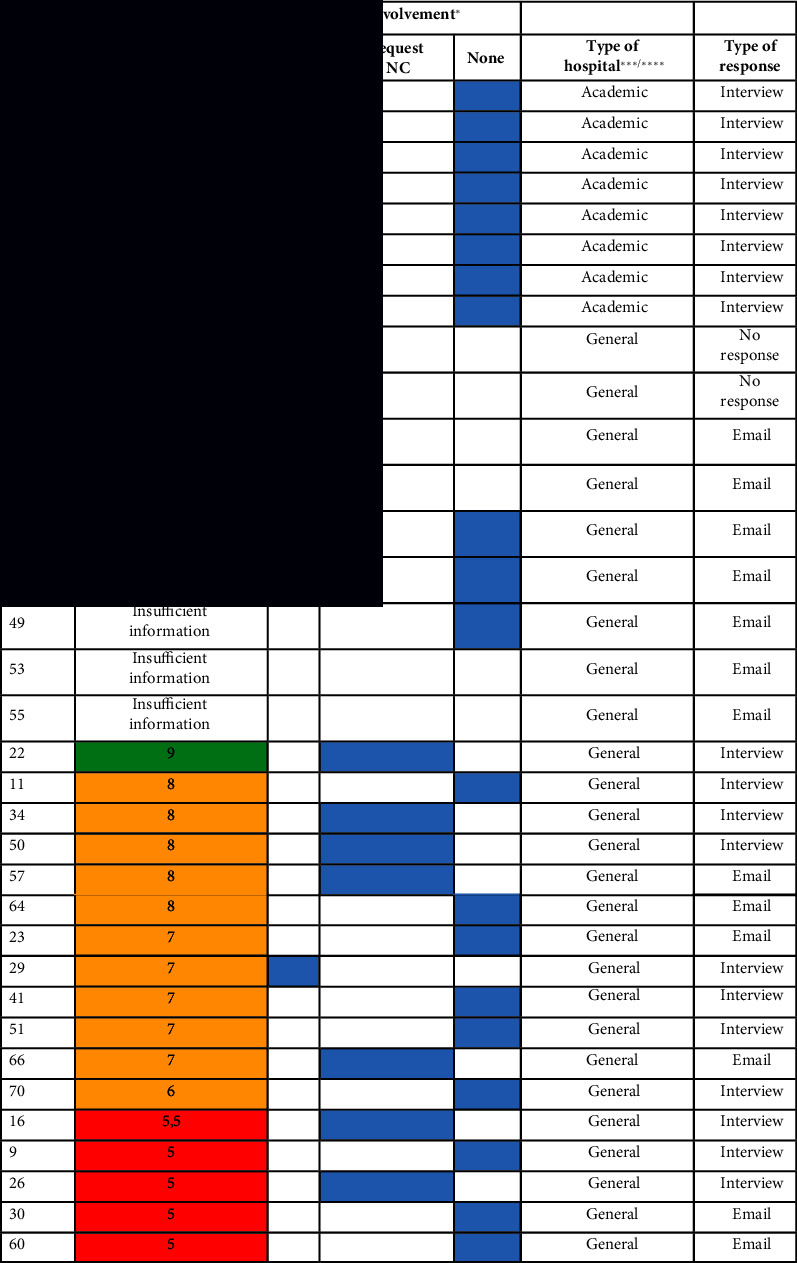
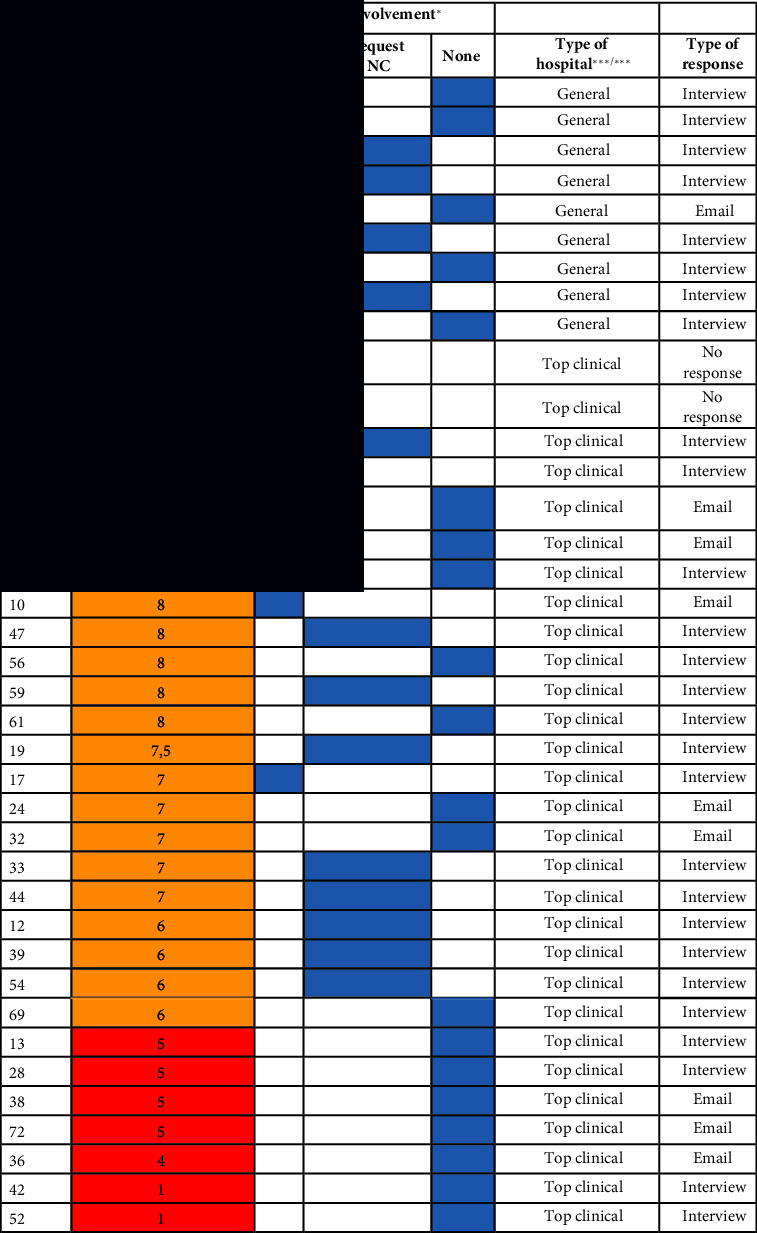

*Note:* ^∗^We asked CNC to pinpoint when their involvement in the crisis management structure of their hospital materialized—from the start of the crisis, on their own request, or not at all. ^∗∗^We asked CNC to score their involvement in the crisis management structure of their hospital on a scale from 1 to 10 (*low* to *high*). ^∗∗∗^Due to a merger, there are seven academic hospitals in the Netherlands since 2018. Nevertheless, the NCs were not merged at the time of the interviews and both were interviewed for our study, hence the eighth academic entry in our dataset. ^∗∗∗∗^Besides academic and general, there are also the so-called top clinical hospitals (26) in the Netherlands. These hospitals have a more specialized infrastructure for complex care than general hospitals (see https://www.stz.nl/over-ons/stz-ziekenhuizen/, visited on the 5th of February 2023). Antoni van Leeuwenhoek and Princes Maxima Centre for (child) oncology are specialized hospitals and are labeled “top clinical” in this overview to warrant anonymity.

## Data Availability

The data that support the findings of this study are available on request from the corresponding author. The data are not publicly available due to privacy or ethical restrictions.

## Appendices

### A. Interview Questions

1. Are you as nurse and/or NC involved in corona crisis management? Please elaborate.
2. On what levels of the organization are you, as NC, involved? Operational, strategic, different.
3. How are you/the NC attributing to the crisis management?
4. In what way, and by whom, is the NC asked to contribute to crisis management? Please elaborate on the actions of the NC itself to contribute.
5. How content are you—on a scale from 1 to —with the involvement of the NC in crisis management? Please elaborate.
6. What are the tasks of nurses and NC in crisis management, and can you elaborate on to what extent nurses and members of the NC are equipped to execute these tasks?
7. What are your learning experiences this far on working in crisis management?
8. How do other agents (managers, physicians, board members, nurses) in the hospital perceive and value the contribution of nurses/NC to the crisis management?
9. How did working in crisis influence the involvement and/or positioning of the NC in the hospital? Please elaborate.
10. What was observed as “nurse leadership” during crisis? Please share your observations.
11. How are nurses/NC involved in the transition from crisis management back to postcrisis organization of care processes?

### B. Topic Guide Focus Groups

Topic guide focus group meetings—collaboration in times of crisis is described in Table B1.

### C. Overview of Involvement Score and Start According to (C)NC, per Type of Hospital and Data Collection Method

Overview of involvement score and start according to (C)NC, per type of hospital, and data collection method is described in Table C1.
